# PKA: assembly of dynamic macromolecular signaling

**DOI:** 10.1186/1471-2210-11-S1-O12

**Published:** 2011-08-01

**Authors:** Susan S Taylor, Ronit Ilouz, Manjula Darshi, Ganapathy Sarma, Alexandr Kornev, Ping Zhang

**Affiliations:** 1 Chemistry/Biochemistry and Pharmacology, University of California, San Diego, USA

## 

Although we have learned a great deal about the protein kinase superfamily from our structure/function studies of the free PKA catalytic (C) subunit and the free cAMP-bound regulatory (R) subunits, we did not understand how the C-subunit was inhibited in the holoenzyme complexes, nor did we understand at the molecular level how the holoenzymes were activated by cAMP. There are four functionally non-redundant R-subunits (RIα, RIβ, RIIα, and RIIβ) and each is assembled as a dimer. The N-terminal dimerization/docking (D/D) is fllowed by a flexible linker and two tandem camp binding domains. The D/D domain also serves as the docking site for A Kinase Anchoring Proteins (AKAPs) which serve as scaffolds that target the PKA holoenzyme to specific sites in the cell in close proximity to specific substrates. Isoform diversity of the R-subunits is a major mechanism for achieving specificity in PKA signaling. We describe here, for the first time, how PKA is assembled into diverse tetrameric holoenzymes and this allows us to begin to fully appreciate the role of the regulatory subunits in creating novel tetramers that are then recruited in unique ways to supramolecular complexes. How these scaffolds are then assembled as part of polyvalent PKA signaling scaffolds is our next challenge. By showing how the RII subunit is anchored to a PDZ domain through a dual specific AKAP, DAKAP2, we are beginning to understand how larger scaffolds are assembled and anchored to the C-termini of ion transporters. We are also exploring PKA signaling scaffolds at the mitochondria.

